# iVAC-XS15-CLL01: personalized multi-peptide vaccination in combination with the TLR1/2 ligand XS15 in CLL patients undergoing BTK-inhibitor-based regimens

**DOI:** 10.3389/fonc.2024.1441625

**Published:** 2024-08-26

**Authors:** Alexander Englisch, Clara Hayn, Susanne Jung, Jonas S. Heitmann, Christopher Hackenbruch, Yacine Maringer, Annika Nelde, Marcel Wacker, Monika Denk, Lisa Zieschang, Christine Kammer, Peter Martus, Helmut R. Salih, Juliane S. Walz

**Affiliations:** ^1^ Clinical Collaboration Unit Translational Immunology, German Cancer Consortium (DKTK), Department of Internal Medicine, University Hospital Tübingen, Tübingen, Germany; ^2^ Department of Peptide-based Immunotherapy, University and University Hospital Tübingen, Tübingen, Germany; ^3^ Department of Obstetrics and Gynecology, University Hospital Tübingen, Tübingen, Germany; ^4^ Cluster of Excellence iFIT (EXC2180) “Image-Guided and Functionally Instructed Tumor Therapies”, University of Tübingen, Tübingen, Germany; ^5^ Institute for Clinical Epidemiology and Applied Biometry, University Hospital Tübingen, Tübingen, Germany

**Keywords:** chronic lymphocytic leukemia, peptide vaccine, BTK-inhibitor, minimal residual disease, immunotherapy, phase I trial, personalized medicine

## Abstract

Chronic lymphocytic leukemia (CLL) is the most common form of leukemia among adults in Western countries. Despite the introduction of targeted therapies, including first-line Bruton’s tyrosine kinase inhibitor (BTKi) treatment, CLL remains largely incurable. Frequent disease relapses occur due to remaining treatment-resistant CLL cells, calling for novel therapies to eliminate minimal residual disease (MRD). Peptide-based vaccination targeting human leucocyte antigen (HLA)-presented CLL-associated antigens represents a promising, low-side-effect therapeutic option to optimize treatment responses and eliminate residual tumor cells by inducing an anti-leukemic immune response. The iVAC-XS15-CLL01 trial is an open-label, first-in-human (FIH) Phase I trial, evaluating the CLL-VAC-XS15 vaccine in CLL patients undergoing BTKi-based therapy. The vaccine was developed from HLA-presented CLL-associated antigen peptides, identified through comparative mass-spectrometry-based immunopeptidome analyses of CLL versus healthy samples in a previous study. To facilitate rapid and cost-effective deployment, vaccine peptides are selected for each patient from a pre-manufactured “peptide warehouse” based on the patient’s individual HLA allotype and CLL immunopeptidome. The trial enrolls 20 CLL patients, who receive up to three doses of the vaccine, adjuvanted with the toll-like-receptor (TLR) 1/2 ligand XS15 and emulsified in Montanide ISA 51 VG. The primary objective of the iVAC-XS15-CLL01 trial is to assess the safety and immunogenicity of the CLL-VAC-XS15 vaccine. Secondary objectives are to evaluate the vaccine impact on MRD, progression-free survival, and overall survival, as well as comprehensive immunophenotyping to characterize vaccine-induced T-cell responses. This Phase I trial aims to advance CLL treatment by enhancing immune-mediated disease clearance and guiding the design of subsequent Phase II/III trials to implement a new therapeutic strategy for CLL patients.

## 1 Introduction

Chronic lymphocytic leukemia (CLL) is the most common leukemia among adults and occurs mainly in elderly people with a median age at diagnosis of 70 years (1, 2). Treatment options for CLL have improved significantly over the past decade. The shift from traditional chemoimmunotherapy to the use of targeted inhibitors has been facilitated by a deeper understanding of the disease’s pathophysiology, and novel targeted therapies now represent the standard of care (3). Bruton’s tyrosine kinase inhibitor (BTKi) based regimens, along with the combination of the B-cell lymphoma 2 (BCL-2) inhibitor Venetoclax, and Obinutuzumab, are the preferred options for first-line treatment in CLL patients (4). However, despite initial response rates of over 80%, most therapies achieve only partial responses, and none of the currently available therapies achieves a cure of the disease, necessitating continuous treatment and bearing the risk of treatment resistance and the accumulation of side effects (5). Persisting residual CLL cells, so-called minimal residual disease (MRD), mediate disease relapse (6). Therefore, the successful elimination of MRD is crucial for achieving long-lasting remission and a potential cure for CLL patients.

Scientific evidence from the success of allogeneic stem cell transplantation (7, 8), spontaneous remissions after viral infections (9) and the generally heterogeneous disease course of CLL (10), points to an immune control of the disease that may be strengthened or induced by immunotherapeutic approaches. Antigen-specific immunotherapies, particularly peptide-based vaccines targeting tumor-associated human leukocyte antigen (HLA)-presented peptides, represent a promising, low-side-effect strategy for inducing anti-tumor T-cell responses. So far, the broad application of peptide-based vaccines in cancer patients is hindered by the challenges of time- and cost-intensive personalized vaccine design, as well as a lack of neoepitopes from tumor-specific mutations. This complicates the identification of target structures through genome-based sequencing approaches, especially in low-mutational burden malignancies like CLL (11).

To overcome these limitations, we have developed a mass-spectrometry-based immunopeptidome-guided workflow for the design of a tumor-associated, off-the-shelf peptide warehouse for broadly applicable personalized vaccines (12–14). This workflow facilitates the identification of tumor-specific HLA-ligands, including non-mutated tumor peptides that arise from altered gene expression or protein processing in tumor cells. By immunopeptidome analyses of primary CLL samples and comparison with an extensive dataset of benign tissues, we recently identified highly frequent, non-mutation-derived, CLL-associated antigens (12). These CLL-associated peptides were further shown to be recognized by pre-existing and *de-novo* induced T-cells in CLL patients. The number of presented CLL-associated HLA peptides, and their recognition by patients T-cells positively correlate with disease outcome, confirming the pathophysiological relevance of these antigens (15).

We here report the trial protocol of the first-in-human (FIH) phase I trial iVAC-XS15-CLL01, which evaluates the personalized multi-peptide vaccine CLL-VAC-XS15, based on CLL-associated peptides. The most frequent CLL-associated peptides were compiled in a premanufactured peptide warehouse, allowing personalized vaccine cocktails tailored to each patient’s HLA allotype and immunopeptidome analysis. The ability to personalize the vaccine cocktail based on the immunopeptidome distinguishes iVAC-XS15 from most previous CLL vaccines (NCT03219450, NCT03939234 (16)) and ensures that the target antigens are naturally presented on CLL cells. While current vaccinations are limited to one or a few peptides restricted to one HLA allotype, iVAC-XS15 contains multiple peptides restricted to both HLA class I and HLA class II. Previous vaccination trials in CLL patients have shown impaired cellular immune responses in CLL patients (17, 18). The iVAC-XS15-CLL01 trial addresses this issue and builds on our previous vaccination trials in immunocompromised patients NCT04954469 (19) by incorporating the new and innovative adjuvant XS15. XS15 is a water-soluble derivative of Pam3Cys. Emulsified in Montanide ISA 51 VG, XS15 has demonstrated safety, tolerability, and has proven to induce strong and long-lasting T cell responses even in immunocompromised patients (20, 21).

## 2 Methods and analysis

### 2.1 Trial design

**Figure 1:**
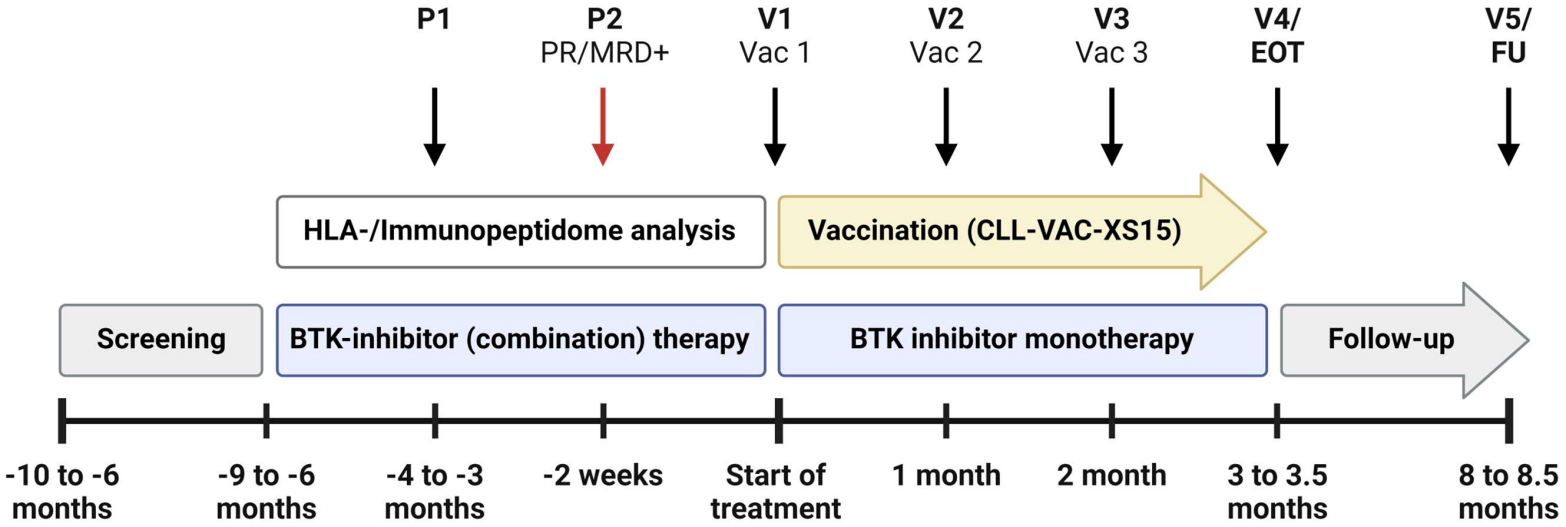
Treatment schedule of the iVAC-XS15-CLL01 trial. The schedule begins with two pre-vaccination visits (P1, P2) and includes five trial visits (V1-V5) for peptide-based vaccination (Vac1-Vac3), followed by an end-of-treatment visit (EOT) and a follow-up visit (FU). Key milestones, such as achieving at least partial remission (PR) and detecting minimal residual disease positivity (MRD+) at P2, are highlighted. The schedule permits the integration of pre-vaccination therapies with Bruton’s tyrosine kinase (BTK) inhibitors and anti-CD20 therapies like Obinutuzumab, as approved by the European Medicines Agency (EMA). Created with BioRender.com.

iVAC-XS15-CLL01 is an open-label, FIH Phase I trial evaluating the safety, immunogenicity, and preliminary efficacy of the CLL-VAC-XS15 personalized multi-peptide vaccine in CLL patients undergoing BTKi-based standard therapy. Patients with prior exposure to BCL-2 inhibitors or chemoimmunotherapy are excluded from the trial. After an initial screening phase, only patients who achieve at least a partial remission (PR) under BTKi-based standard therapy and show MRD positivity in blood or bone marrow enter the treatment phase and receive the CLL-VAC-XS15 vaccine. These patients represent the trial cohort. The trial duration for each patient is approximately 10 months starting with the first vaccination, including a four-month treatment phase and a six-month follow-up period. The overall duration of the trial is expected to be approximately 3 years. Patients progress through the following trial phases (**Figure 1**):

#### 2.1.1 Screening

**Figure 2:**
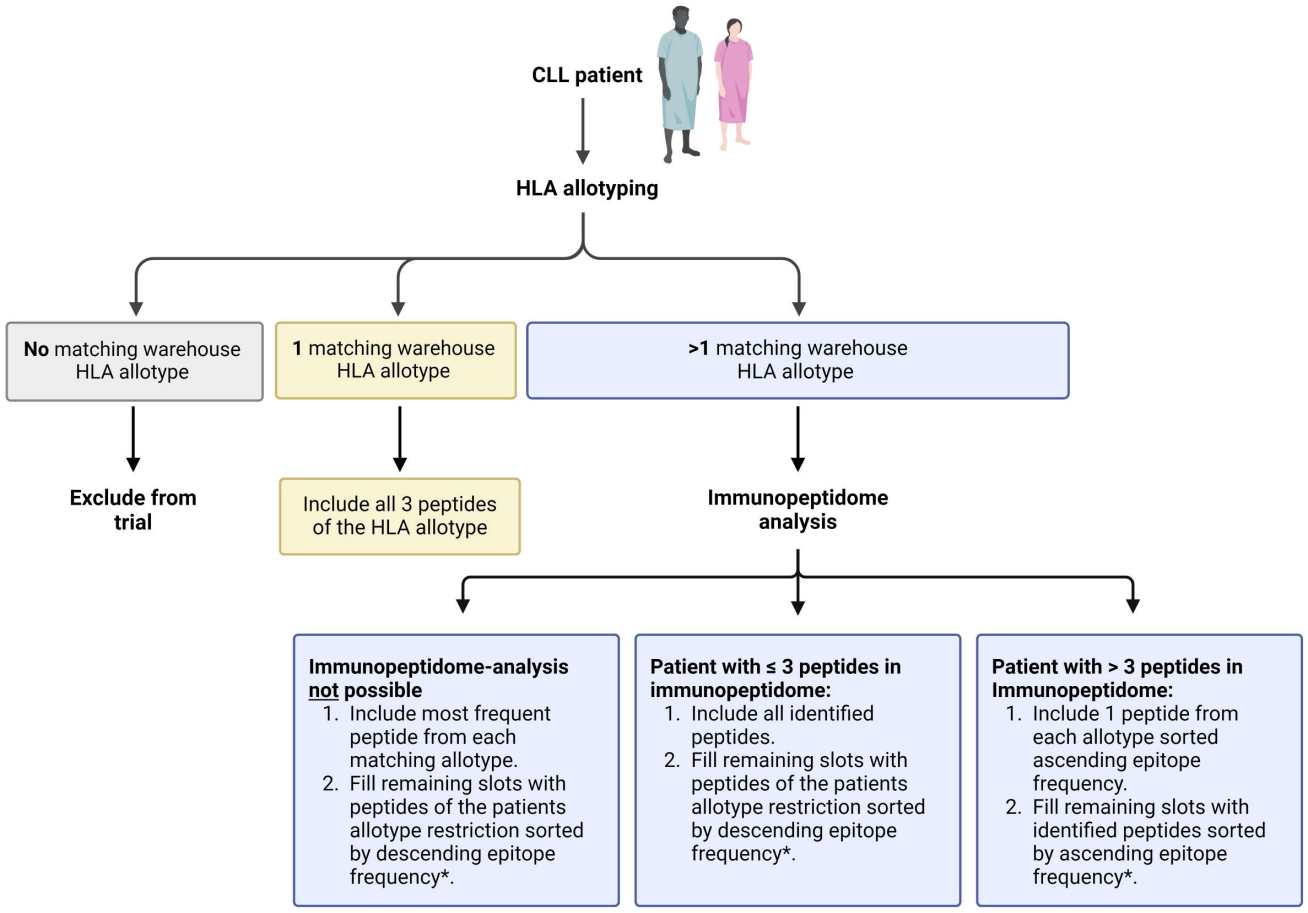
Process of patient-individual vaccine composition. The CLL-VAC-XS15 vaccine comprises a personalized multi-peptide cocktail tailored for each patient based on their human leukocyte antigen (HLA) allotype and, if applicable, an immunopeptidome analysis performed on peripheral blood mononuclear cells (PBMCs). Using a predefined algorithm, HLA class I-restricted vaccination peptides are selected from pre-produced warehouse peptides. HLA class II-restricted vaccination peptides include chronic lymphocytic leukemia (CLL)-specific peptides, which are added to the peptide cocktail independently of the patient’s HLA allotype (purple), along with control peptides (white). Peptides are combined with the adjuvant XS15 and emulsified in Montanide, forming the individual CLL-VAC-XS15 vaccine for the patient. Created with BioRender.com.

**Figure 3:**
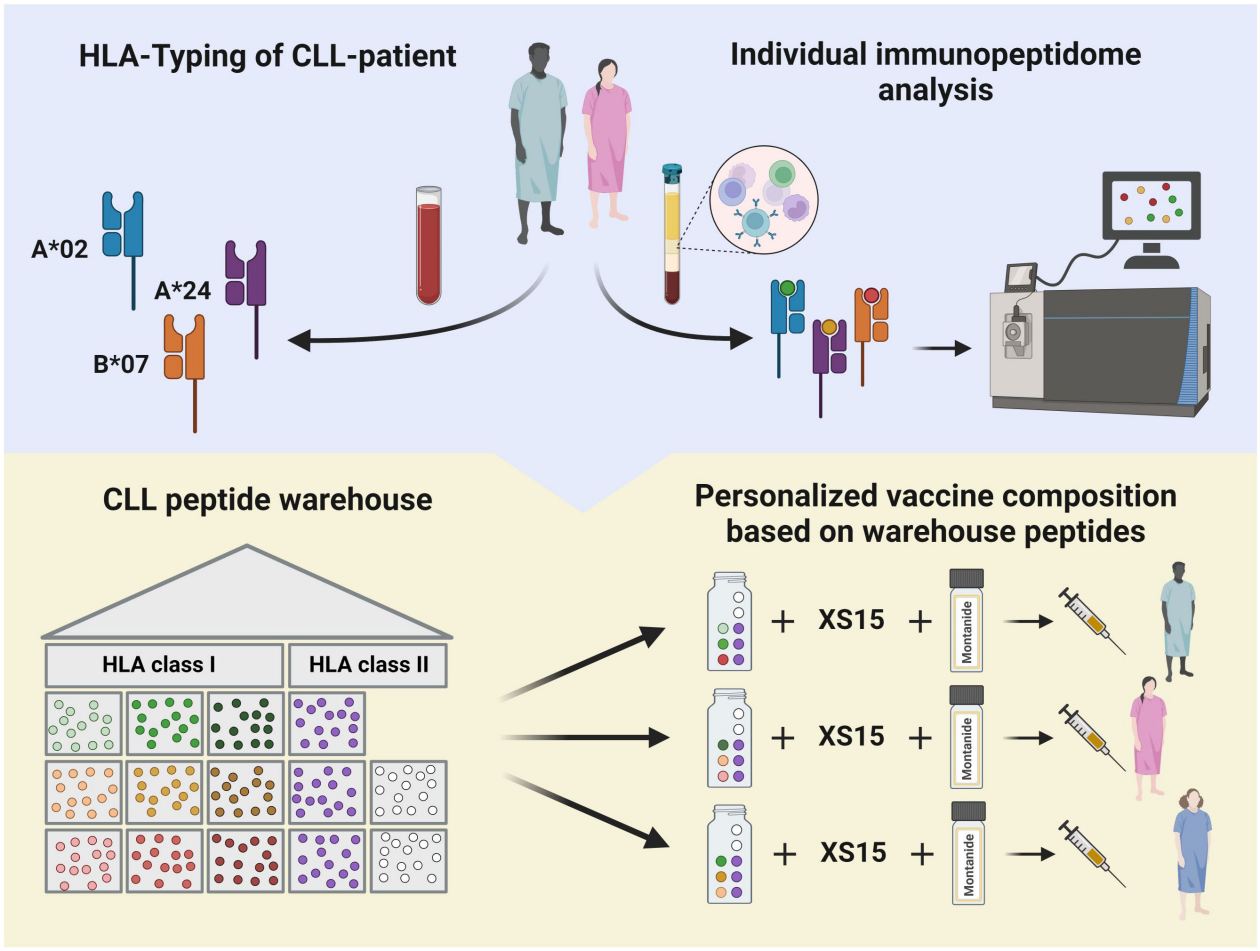
Detailed outline of the peptide selection algorithm for human leukocyte antigen (HLA) class I-restricted CLL-associated peptides. Peptide selection depends on the patient’s HLA allotype and immunopeptidome analysis. *randomized peptide selection if frequencies of two peptides are identical. Created with BioRender.com.

Informed consent is acquired from participants who enter the screening process. Eligibility of patients is determined through initial evaluation of inclusion and exclusion criteria. The assessment involves evaluating the CLL disease state, reviewing the planned BTKi therapy, and assessing any concurrent medications. HLA allotyping and CLL-specific immunopeptidomic analyses are initiated to enable personalized warehouse-based vaccine composition based on a predefined algorithm (**Figures 2**, **3**).

#### 2.1.2 BTKi treatment

After screening, patients start treatment with a BTKi-based therapy. Combination therapies approved by the EMA, such as anti-CD20 antibody treatment (e.g. Obinutuzumab), are permitted. Two pre-vaccination visits (P1 and P2) are scheduled:

- P1: Three to four months after BTKi treatment initiation, to assess therapy response according to the International Workshop on Chronic Lymphocytic Leukemia (iwCLL) guidelines (22) and MRD in peripheral blood.
- P2: Four to six weeks after the last application of combination therapy, or, if BTKi is applied as single agent, six to eight months after treatment initiation, to assess treatment response according to iwCLL guidelines (22) and MRD in the peripheral blood and bone marrow. Vaccination must start within two weeks after P2.

#### 2.1.3 Vaccination phase

Patients, who achieved at least a PR (22) with proof of MRD positivity in blood or bone marrow at P2, are vaccinated six to nine months after starting BTKi treatment. Patients who are MRD negative or have not achieved a PR at P2 do not enter the treatment phase and are considered as screening failures. Combination anti-CD20 therapy must be completed before the start of vaccination. BTKi treatment is continued during the vaccination phase. Each patient receives a maximum of three vaccinations, at four-week intervals. The vaccine is applied subcutaneously at the abdomen. The site of vaccination (either right or left) is retained for all vaccinations. Patients are monitored for at least two hours after each vaccination to ensure immediate care in case of severe or unexpected adverse events, particularly allergic reactions. An end-of-treatment (EOT) visit is conducted 4 to 6 weeks after the final vaccination.

#### 2.1.4 Follow-up

A follow-up visit is scheduled six to six-and-a-half months after EOT to assess clinical and immunological efficacy and to collect comprehensive safety data.

### 2.2 Trial population

The trial population comprises 20 patients with a confirmed diagnosis of CLL according to the iwCLL guidelines and positivity for one of the following HLA alleles: HLA-A*02, A*24, B*07 (22). Patients must meet the inclusion criteria outlined below at the time of screening and must not fulfill any of the exclusion criteria. Before entering the vaccination phase, a re-evaluation is conducted, and additional inclusion and response criteria must be met. The trial population includes both genders, with no predefined quantitative ratio between females and males.

#### 2.2.1 Inclusion criteria

##### 2.2.1.1 Inclusion criteria at screening

- Age ≥ 18 years.
- Ability to understand and willingness to sign a written informed consent form.
- Ability to adhere to the trial schedule and other protocol requirements.
- Eastern Cooperative Oncology Group (ECOG) performance status score of ≤ 2.
- Documented diagnosis of CLL according to iwCLL guidelines (22).
- CLL that warrants treatment according to modified criteria for initiation of therapy (22):
  - Massive (i.e., lower edge of spleen ≥ 6 cm below the left costal margin), progressive, or symptomatic splenomegaly, or
  - Massive (i.e., ≥ 10 cm in the longest diameter), progressive, or symptomatic lymphadenopathy, or
  - Progressive lymphocytosis in the absence of infection, with an increase in blood absolute lymphocyte count ≥ 50% over two months or lymphocyte doubling time of < six months, or
  - Autoimmune anemia and/or thrombocytopenia that is poorly responsive to corticosteroids or other standard therapy, or
  - Constitutional symptoms, defined as any one or more of the following disease-related symptoms or signs occurring in the absence of evidence of infection:
    ▪ Unintentional weight loss of ≥ 10% within the previous 6 months, or
    ▪ Significant fatigue (≥ grade 2), or
    ▪ Fevers > 38.0°C for ≥ 2 weeks, or
    ▪ Night sweats for > 1 month.
- Planned initiation of a BTKi-based monotherapy or combination therapy (e.g. anti-CD20-based regimens).

##### 2.2.1.2 Inclusion criteria for entering the vaccination phase

- HLA typing positive for any of the following alleles: HLA-A*02 or A*24, B*07.
- Prior BTKi treatment of at least 6 months and no longer than 8 months.
- Ongoing BTKi monotherapy.
- Achievement of response [at least PR according to iWCLL guidelines (22)].
- MRD positivity (CLL cells in peripheral blood ≥ 10^-4^ determined by flow cytometry).
- Negative SARS-CoV-2 test, as long as requested by state regulations.
- Negative serological Hepatitis B test or negative PCR in case of positive serological test without evidence of an active infection, negative testing of Hepatitis C RNA, negative HIV test within 6 weeks before trial inclusion.
- Female patients of childbearing potential (FCBP) and male patients with partners of childbearing potential who are sexually active must agree to use two effective forms of contraception throughout the trial. Additionally, FCBPs must provide a negative urine or serum pregnancy test within 14 days before vaccination.
- Postmenopausal or evidence of non-childbearing status defined as:
  - Amenorrhoeic for 1 year or more following cessation of exogenous hormonal treatments.
  - Luteinizing hormone (LH) and Follicle-stimulating hormone (FSH) levels in the post-menopausal range for women under 50 years.

#### 2.2.2 Exclusion criteria

- Pregnant or lactating females.
- Planned treatment regime without BTKi.
- Adverse events related to BTKi therapy graded > 2 according to the Common Toxicity Criteria for Adverse Events (CTCAE) version 5.0.
- Participation in any clinical trial or having taken any investigational therapy that would interfere with the trial’s primary and secondary endpoints.
- Prior history of malignancies other than CLL, unless the subject has been free of the disease for ≥ 5 years. Exceptions include the following:
  - Basal cell carcinoma of the skin.
  - Carcinoma *in situ* of the cervix.
  - Carcinoma *in situ* of the breast.
  - Incidental histological finding of prostate cancer (TNM stage of T1a or T1b).
- Disease transformation (active), i.e. Richter’s syndrome, prolymphocytic leukemia.
- Autoimmune hemolysis or immune thrombocytopenia caused by CLL.
- Any immunosuppressive treatment not related to CLL except corticosteroids.
- Pre-existing auto-immune disease except for Hashimoto thyroiditis and mild (not requiring immunosuppressive treatment) psoriasis.
- Chronic lung disease requiring drug treatment.

### 2.3 Investigational drug

The CLL-VAC-XS15 vaccine is designed as a personalized multi-peptide cocktail containing six CLL-associated peptides and two control peptides selected from premanufactured CLL warehouse peptides, adjuvanted with the TLR1/2 ligand XS15. Before administration, the peptide cocktail is emulsified in a 1:1 water-to-oil emulsion with Montanide ISA 51 VG. Each peptide in the cocktail is administered at a dose of 300 µg, determined to be effective in inducing robust and durable T-cell responses (19, 21, 23). The TLR1/2 ligand XS15 is included at a dose of 50 µg and is dissolved in a 33% DMSO and water solution, a formulation that has proven to be safe and well-tolerated in previous trials (19, 21, 23).

The vaccine peptides for each patient are selected from a premanufactured peptide warehouse comprising nine CLL-associated HLA class I-restricted peptides (3 x HLA-A*02, 3 x HLA-A*24, 3 x HLA-B*07) and three CLL-associated HLA class II-restricted peptides. These warehouse peptides were defined by mass-spectrometry-based immunopeptidomics in a large cohort of primary CLL samples and were selected based on their frequent presentation on CLL cells compared to normal tissues and their ability to elicit functional T-cell responses (12).

The personalization of the peptide cocktail involves the selection of three CLL-associated HLA class I-restricted peptides based on the patient’s HLA allotype and the immunopeptidome of their CLL cells. Patients with only one corresponding HLA class I allotype receive all three peptides of that type. Those with more than one matching allotype have their peptides tailored based on individual immunopeptidome analyses (**Figure 3**). The three CLL-associated HLA class II-restricted peptides included in the peptide cocktails do not require allotype-based selection due to their promiscuous binding to multiple HLA class II allotypes, ensuring broad compatibility (24).

In addition to CLL-associated peptides, the cocktail contains two HLA class II-restricted control peptides serving dual functions: One peptide, derived from the hexon protein of adenovirus C, assesses the patient’s general ability to mount a T-cell response against non-self antigens (25). The second control peptide, derived from BIRC5/survivin, is a pan-tumor-associated peptide that induces strong CD4^+^ T-cell responses, serving as a reference for evaluating responses induced by the CLL-associated peptides (26, 27).

### 2.4 Vaccination schedule

Patients are scheduled to receive three subcutaneous vaccinations of CLL-VAC-XS15, administered at four-week intervals. This schedule is based on previous clinical experiences with peptide-based vaccines combined with the TLR1/2 ligand XS15 in healthy volunteers and cancer patients (19–21, 23). Observations from our SARS-CoV-2 peptide-based vaccine CoVac-1 indicated a decline in T-cell response twelve months after administration in elderly volunteers (28). However, XS15-adjuvanted vaccines in cancer patients maintained strong T-cell responses for more than two years after just two doses (21). Even highly immunocompromised patients achieved strong immune responses with no more than three doses (21). Thus, given the advanced age and immunosuppressed status of CLL patients, a three-dose schedule has been adopted to ensure adequate immunogenicity. The decision to proceed with subsequent vaccinations is based on the patient’s tolerance of previous doses without significant toxicity. For CTCAE grade 3 or higher adverse events or the presence of skin ulcerations, vaccinations may be postponed for up to four weeks or until the event resolves to grade 2 or lower or ulcerations resolve. For CTCAE grade 4 events, further vaccinations will be permanently discontinued unless the event is clearly unrelated to the vaccine. No premedication is required prior to vaccination.

### 2.5 Trial objectives

The iVAC-XS15-CLL01 trial evaluates the safety, immunogenicity, and preliminary efficacy of the CLL-VAC-XS15 personalized multi-peptide vaccine administered together with standard BTKi-based therapy in CLL patients.

#### 2.5.1 Primary objectives

The trial is designed to evaluate two primary objectives:

- The primary safety objective is to determine the safety and tolerability of the CLL-VAC-XS15 vaccine. This is systematically determined by monitoring the frequency and severity of adverse events (AEs), serious adverse events (SAEs), and suspected unexpected serious adverse reactions (SUSARs) according to the CTCAE V5.0 guidelines.
- The primary efficacy objective is to assess the immunogenicity of the CLL-VAC-XS15 vaccine by evaluating the induction of peptide-specific T-cell responses. These responses are quantified using Interferon-gamma (IFN-γ) Enzyme-linked Immunosorbent Spot (ELISPOT) assays at various stages of the trial (visits 2, 3, 4, and follow-up visit) compared to baseline levels. Patients are considered analyzable when they have received at least two vaccinations, and data on immunogenicity is available.

#### 2.5.2 Secondary objectives

The secondary objectives of this trial are:

##### 2.5.2.1 To analyze MRD negativity

MRD negativity in peripheral blood is defined as the presence of less than one CLL cell among 10,000 leukocytes (0.01%) and is measured by flow cytometry at the EOT and during follow-up.

##### 2.5.2.2 To evaluate progression-free survival and overall survival

PFS is the time from the first vaccination to the progression of disease or death from any cause, whichever comes first. OS is the time from the first vaccination until death from any cause. Patients are censored at their last follow-up.

##### 2.5.2.3 To assess the duration of response

DOR is measured from the date of first documented response to the first occurrence of progression or relapse (determined according to the standard iwCLL guidelines) or death by any cause, whichever occurs first. Patients are censored at their last follow-up.

##### 2.5.2.4 To evaluate the quality of life during trial treatment

QoL is measured using the overall quality of life scores from the European Organization for Research and Treatment of Cancer Quality of Life Questionnaire Core 30 (EORTC QLQ-C30). Assessments occur at visit 1, EOT, and during follow-up.

##### 2.5.2.5 To assess changes in lymphocyte immunophenotypes

Absolute changes in the number and percentage of lymphocyte subsets (B-cell subsets, T-cell subsets, NK-cells) and myeloid-derived suppressor cells (MDSC) are analyzed from pre-vaccination visit two P2 (baseline) through follow-up using flow cytometry.

##### 2.5.2.6 To characterize the vaccine-induced T-cell response

The vaccine-induced T-cell responses are characterized using flow-cytometry-based phenotyping and functionality testing. This analysis includes assessing peptide-specific T-cells collected from the pre-vaccination visit P2 (defined as baseline) and subsequent samples obtained during follow-up visits.

### 2.6 Safety

Safety and toxicity of the CLL-VAC-XS15 vaccine are evaluated based on the CTCAE V5.0 guidelines and assessed descriptively (29). To ensure comprehensive safety monitoring, serial measurements are conducted at screening and scheduled intervals throughout the trial. All AEs and SAEs are documented and reported according to Good Clinical Practice (GCP) guidelines. Any SAEs that are both suspected (potentially related to the vaccine) and unexpected (not consistent with the known properties of the product) are classified as SUSARs and require expedited reporting to the responsible ethics committees, the Paul-Ehrlich-Institut (PEI) as the competent higher federal authority, and to all participating investigators.

### 2.7 Data safety monitoring board

An independent Data and Safety Monitoring Board (DSMB) consisting of experts in hematology, oncology, and immunology, monitors the trial’s progress, safety data, and key efficacy endpoints. The DSMB ensures ethical trial conduct and patient safety and receives regular updates from the Sponsor and/or Clinical Investigator about all safety-related events. Before the start of the trial and annually thereafter, the DSMB receives a report summarizing all relevant safety data, recruitment rates, and trial status. In case of safety concerns, such as vaccine-related (SAEs), the DSMB can convene an emergency meeting. The DSMB provides recommendations on whether to modify, continue, or terminate the trial based on its reviews.

### 2.8 Sample size calculation

The sample size was established based on statistical power considerations and type I error probability. Assuming a peptide-specific immune response in fewer than 30% of patients, the trial is designed to limit the type I error (false positive rate) to 5%, preventing premature advancement of ineffective therapy. For a response in 60% or more of patients, the trial aims for a statistical power of 80%, ensuring a high likelihood of correctly identifying effective treatment worthy of Phase III trials. The calculations are based on a binomial distribution with a sample size of 20 patients, achieving an exact power of 87% and a type I error of 4.8%. A minimum of 10 patients must demonstrate an immune response to consider the vaccine for further clinical development.

## 3 Discussion

The iVAC-XS15-CLL01 trial is a FIH trial evaluating the safety and immunogenicity of a personalized peptide-based vaccination adjuvanted with the TLR1/2 ligand XS15 in CLL patients under BTKi-based treatment. The multi-peptide vaccine CLL-VAC-XS15 comprises naturally presented non-mutated CLL-associated antigens expected to induce multiple leukemia-specific CD8^+^ and CD4^+^ T-cell responses that lead to the killing of residual CLL cells and thus protection from disease relapse, eventually resulting in prolonged survival.

Despite numerous previous clinical trials, which have often failed to translate robust T-cell responses into tangible clinical benefits (30–36), we and others provided compelling evidence that T-cell responses against HLA-presented tumor antigens are associated with improved clinical outcomes in several malignancies, including CLL (15, 37–39). This points to a potential for clinical efficacy of peptide-based vaccines in CLL, presuming their design and application are meticulously optimized. An additional challenge in the development of peptide-based vaccines represents the time-consuming and costly process of producing individualized vaccine products (11). The iVAC-XS15-CLL01 trial aims to overcome these limitations by addressing several key prerequisites for clinical effective vaccine design:

A central component in vaccine development is the selection of optimal target antigens. These targets consist of HLA-presented peptides that are naturally, frequently, and exclusively presented on the surface of malignant cells, eliciting a specific T-cell immune response. The discrepancy between gene expression and HLA-restricted presentation of these antigens necessitates the use of direct methods to identify potential vaccine targets. Mass-spectrometry-based immunopeptidome analyses represent the only unbiased method for the direct identification and characterization of naturally presented tumor-associated peptides on the cell surface of malignant cells (40, 41). In recent years, we used this approach to identify and characterize tumor-associated antigen peptides in various hematological malignancies, including CLL, and proved their pathophysiological relevance for disease outcome (12, 15, 39, 42–44).

To address the challenge of the time- and cost-intensive production of personalized vaccines, we have implemented a “peptide warehouse concept” to streamline this process (12, 45). This approach involves the premanufacturing of highly frequent tumor-associated antigens, which are then used to formulate personalized vaccine cocktails based on the patient’s HLA allotype and immunopeptidome analyses. The iVAC-XS15-CLL trial employs such a peptide warehouse specifically designed for CLL. While the selection of HLA class I-restricted peptides for the CLL-VAC-XS15 vaccine must be personalized, the HLA class II-restricted peptides included in the vaccine cocktail can be administered to all patients due to their promiscuous binding to multiple HLA class II allotypes (23, 45). By targeting multiple antigens per patient, the risk of immune escape due to the loss of antigens is minimized (46).

Besides the selection of optimal antigen targets, a key prerequisite for efficient peptide vaccination is the usage of a potent adjuvant enabling the induction of strong and long-lasting immune responses. In the iVAC-XS15-CLL01 trial, we apply an innovative adjuvant formulation based on the TLR1/2 agonist XS15 that has already proven safety and tolerability as well as potent T cell activation in clinical trials (NCT04546841 (23), NCT04954469 (19)). XS15, a water-soluble derivative of the TLR1/2 ligand Pam3Cys (20), is combined with the peptide cocktail and emulsified with Montanide ISA 51 VG, resulting in an oily vaccine formulation for subcutaneous injection into the abdominal tissue. This formulation is designed to provide continuous immune stimulation without systemic side effects and has been shown to induce potent CD8^+^ and Th1 CD4^+^ T-cell responses after single dosing, exceeding those elicited by other peptide and mRNA-based vaccines (20). In addition, the specific XS15 adjuvant formulation creates a depot at the injection site where the vaccinated peptides persist, thereby facilitating the continuous induction of robust immune responses lasting up to several years (19–21, 23, 28). Importantly, no significant systemic side effects have been reported in healthy volunteers [NCT04546841 (23)] or in cancer patients (21), in particular no allergic or anaphylactic reactions or immune-related side effects.

Alongside optimizing the vaccine design, the rational combination of peptide-based vaccines with established standard therapies is crucial for enhancing their clinical effectiveness. In the iVAC-XS15-CLL01 trial, peptide-based vaccination is administered to patients undergoing BTKi-based treatment who have achieved at least a PR. BTKi therapy significantly reduces tumor mass and restores the T-cell compartment (4, 5). This improves the effector-to-target cell ratio, enabling more CLL-specific T-cells to effectively target and eliminate a smaller number of remaining tumor cells. Furthermore, several studies have shown a positive effect of BTKi on T-cell responses in CLL via enhanced T-cell diversity and effector function as well as improved antigen presentation, and reduced expression of programmed cell death protein 1 (PD1) and programmed cell death ligand 1 (PDL1) of leukemia cells (47–53).

Taken together, the iVAC-XS15-CLL01 trial integrates several innovative strategies to optimize peptide-based immunotherapy for eradication of MRD in CLL. By employing an optimized selection of target antigens based on immunopeptidome analysis, incorporating the innovative adjuvant XS15, and utilizing a peptide warehouse approach for rapid and cost-effective personalized vaccine production, this trial aims to maximize the therapeutic potential of peptide-based vaccination. Furthermore, the combination with effective BTKi therapy enhances the T-cell response and improves the effector-to-target ratio, thus increasing the overall effectiveness of the treatment. While the primary objective of the iVAC-XS15-CLL01 trial is to establish the safety and immunogenicity of the CLL-VAC-XS15 vaccine, the secondary endpoints will provide first evidence for clinical efficacy by assessing MRD elimination and improvement of disease outcome in CLL patients.
